# CD34⁺细胞回输剂量对初诊多发性骨髓瘤患者自体外周血造血干细胞移植后造血重建及移植相关结局的影响

**DOI:** 10.3760/cma.j.cn121090-20260118-00035

**Published:** 2026-06

**Authors:** 蓓蓓 谢, 福润 安, 露露 张, 莉莉 陶, 金晓 侯, 志敏 翟, 毅 董, 慧 秦, 延生 汪

**Affiliations:** 安徽医科大学第二附属医院血液内科，合肥 230601 Department of Hematology, The Second Affiliated Hospital of Anhui Medical University, Hefei 230601, China

**Keywords:** 多发性骨髓瘤, 自体外周血造血干细胞移植, 造血重建, CD34^+^细胞, Multiple myeloma, Autologous peripheral blood stem cell transplantation, Hematopoietic reconstitution, CD34⁺ cells

## Abstract

**目的:**

探讨不同CD34⁺细胞回输剂量对多发性骨髓瘤（MM）患者自体外周血造血干细胞移植（auto-PBSCT）后造血重建及移植相关结局的影响。

**方法:**

回顾性纳入2020年7月27日至2025年8月29日在安徽医科大学第二附属医院首次接受auto-PBSCT的初诊MM患者57例。按回输CD34⁺细胞剂量分为低剂量组（<4×10^6^/kg，19例）与高剂量组（≥4×10^6^/kg，38例）。主要终点为中性粒细胞植入时间及血小板植入时间；次要终点为3～4级中性粒细胞减少及血小板减少持续时间、粒细胞缺乏伴发热持续时间及血小板输注量，并对次要终点进行Benjamini-Hochberg假发现率（FDR）校正。采用Logistic回归分析10 d内中性粒细胞及血小板植入的影响因素。生存结局采用Kaplan-Meier法及Cox回归分析。

**结果:**

57例患者回输CD34⁺细胞量中位数为5.30（3.49，9.35）×10^6^/kg，均获得造血重建。与低剂量组相比，高剂量组中性粒细胞植入时间（中位数：10.00 d对11.00 d，*P*＝0.014）及血小板植入时间（中位数：10.00 d对12.00 d，*P*＝0.009）更短；重度骨髓抑制持续时间更短、粒细胞缺乏伴发热持续时间更短、血小板输注量更少（*P*值均<0.05）。多因素分析显示，CD34^+^细胞回输剂量≥4×10^6^/kg与10 d内中性粒细胞植入独立相关（*OR*＝4.661，95％ *CI*：1.322～16.435，*P*＝0.017）；国际分期系统（ISS）Ⅲ期（*OR*＝0.217，95％ *CI*：0.060～0.781，*P*＝0.019）及移植前化疗次数增加（*OR*＝0.706，95％ *CI*：0.520～0.958，*P*＝0.025）与10 d内血小板植入受限独立相关。生存分析显示，高剂量组无进展生存（PFS）及总生存（OS）优于低剂量组（中位PFS期：未达到对773 d，*P*＝0.040；中位OS期：未达到对812 d，*P*＝0.009），且CD34^+^细胞回输剂量≥4×10^6^/kg可能与较低的复发/进展风险独立相关（*HR*＝0.212，95％ *CI*：0.052～0.870，*P*＝0.031）。

**结论:**

初诊MM患者auto-PBSCT后CD34^+^细胞回输剂量≥4×10^6^/kg与更快的中性粒细胞植入、更短的重度骨髓抑制持续时间和粒细胞缺乏伴发热时间及更少的血小板输注需求相关，并可能改善长期生存预后。

## Abstract (en)

**Objective:**

To investigate the impact of different infused CD34⁺ cell doses on hematopoietic reconstitution and transplant-related outcomes in patients with multiple myeloma（MM）undergoing autologous peripheral blood stem cell transplantation（auto-PBSCT）.

**Methods:**

We retrospectively analyzed 57 newly diagnosed MM patients who underwent first auto-PBSCT at the Second Affiliated Hospital of Anhui Medical University between July 27, 2020 and August 29, 2025. Patients were stratified by infused CD34⁺ cell dose into a low-dose group（<4×10^6^/kg, *n*＝19）and a high-dose group（≥4×10^6^/kg, *n*＝38）. The primary endpoints were time to neutrophil engraftment and time to platelet engraftment. Secondary endpoints included the duration of grade 3–4 neutropenia, duration of grade 3–4 thrombocytopenia, duration of febrile neutropenia, and platelet transfusion requirements. Secondary endpoints were adjusted for multiple comparisons using the Benjamini-Hochberg false discovery rate（FDR）procedure. Logistic regression was used to identify factors associated with neutrophil and platelet engraftment within 10 days. Survival outcomes, including progression-free survival（PFS）and overall survival（OS）, were analyzed using the Kaplan-Meier method and Cox regression analysis.

**Results:**

The median infused CD34⁺ cell dose was 5.30（3.49, 9.35）×10^6^/kg, and all patients achieved hematopoietic reconstitution. Compared with the low-dose group, the high-dose group had shorter time to neutrophil engraftment（median: 10.00 d *vs* 11.00 d, *P*＝0.014）and platelet engraftment（median: 10.00 d *vs* 12.00 d, *P*＝0.009）. The high-dose group also had shorter duration of severe cytopenia, shorter febrile neutropenia duration, and lower platelet transfusion requirements（all *P* value<0.05）. Multivariable analysis showed that an infused CD34⁺ cell dose ≥4×10^6^/kg was independently associated with neutrophil engraftment within 10 days（*OR*＝4.661, 95％ *CI*: 1.322–16.435, *P*＝0.017）, whereas ISS stage Ⅲ（*OR*＝0.217, 95％ *CI*: 0.060–0.781, *P*＝0.019）and a higher number of pre-transplant chemotherapy cycles（*OR*=0.706, 95％ *CI*: 0.520–0.958, *P*＝0.025）were associated with failure to achieve platelet engraftment within 10 days. Survival analysis showed that the high-dose group had superior PFS and OS compared with the low-dose group（median PFS time: not reached vs 773 d, *P*＝0.040; median OS time: not reached *vs* 812 d, *P*＝0.009）, and that an infused CD34⁺ cell dose ≥4×10^6^/kg might be independently associated with a lower risk of relapse or disease progression（*HR*＝0.212, 95％ *CI*: 0.052–0.870, *P*＝0.031）.

**Conclusion:**

In patients with newly diagnosed MM undergoing auto-PBSCT, an infused CD34⁺ cell dose ≥4×10^6^/kg was associated with faster neutrophil engraftment, shorter duration of severe myelosuppression, reduced time of febrile neutropenia, and decreased need for platelet transfusions, and may improve long-term survival outcomes.

自体造血干细胞移植（auto-HSCT）目前仍是新诊断的适合移植的多发性骨髓瘤（MM）患者的一线巩固治疗手段，移植后造血重建过程直接影响骨髓抑制期的感染及出血风险、支持治疗需求。国际骨髓瘤工作组（IMWG）指南提出的单次移植最低输注剂量为2×10^6^/kg[1]。但在临床实践中，回输剂量的差异可能进一步影响造血重建速度、并发症负担以及远期结局。关于最佳回输剂量的界定存在争议，既往研究多聚焦于CD34⁺细胞数对造血重建速度的单一影响，而关于高剂量CD34⁺细胞对移植期间并发症、血制品消耗及生存预后的综合影响数据仍有限。既往共识指南认定自体移植回输CD34⁺细胞最低可接受剂量为2×10^6^/kg，而（4～6）×10^6^/kg为理想的目标剂量范围[1]。基于上述临床共识与可操作性，本研究预先设定4×10^6^/kg作为分层界值，通过回顾性分析我院57例初诊MM患者的移植数据，探究4×10^6^/kg作为界值的高剂量回输在优化移植过程及结局中的临床价值，为临床制定更优动员与移植策略提供依据。

## 病例与方法

1. 病例：本研究为回顾性队列研究。选取2020年7月27日至2025年8月29日于安徽医科大学第二附属医院行自体外周血造血干细胞移植（auto-PBSCT）的初诊MM患者57例，回顾性收集临床资料。纳入标准：①符合IMWG诊断标准；②首次接受auto-PBSCT；③可获得完整的植入与随访信息。排除标准：①二次/串联移植；②合并其他活动性恶性肿瘤。收集的临床资料包括年龄、性别、疾病分期、诱导治疗方案、动员方案、移植前疾病状态、骨髓抑制分级及持续时间、粒细胞缺乏（粒缺）伴发热时间、血制品输注量、回输CD34⁺细胞数量及单个核细胞（MNC）数量、造血重建时间以及无进展生存（PFS）与总生存（OS）随访结局等。本研究经安徽医科大学第二附属医院医学研究伦理委员会批准（批件号：YX2026-231）。

2. 造血干细胞动员及采集：57例患者动员前结合既往治疗暴露（如来那度胺或抗CD38单抗）、治疗线数、外周血象与骨髓储备进行评估；动员过程中依据外周血动态监测及首次采集量进行调整。57例患者动员方案中采用粒细胞集落刺激因子（G-CSF）单药7例、环磷酰胺+G-CSF 7例、普乐沙福+ G-CSF18例（其中一线使用13例、二线使用5例）、依托泊苷+G-CSF 25例。动员后动态监测外周血CD34⁺细胞计数（采用流式细胞术）及血常规，当计算CD34⁺细胞数>20个/µl时启动外周血造血干细胞单采。

3. 预处理方案及回输：57例患者中，56例采用大剂量美法仑方案预处理，1例采用白消安+环磷酰胺+依托泊苷方案预处理。预处理结束后回输冻存的自体外周血造血干细胞，采集的造血干细胞均在本次移植中全部回输，输注的CD34^+^细胞剂量<4×10^6^/kg定义为低剂量组，≥4×10^6^/kg定义为高剂量组。

4. 支持治疗及并发症处理：移植后骨髓抑制期常规予以G-CSF促进中性粒细胞恢复，重组人血小板生成素（TPO）促进血小板恢复，根据出血及血小板恢复等情况决定是否联合重组人TPO受体激动剂（TPO-RA）。PLT<20×10^9^/L或伴有活动性出血时输注血小板；粒缺期出现发热时，根据感染部位及危险度分层予以抗感染治疗。

5. 骨髓抑制分级与持续时间定义：中性粒细胞减少及血小板减少的分级采用美国国立癌症研究所不良事件通用术语标准（CTCAE）5.0。中性粒细胞减少：3级为ANC<1.0×10^9^/L且≥0.5×10^9^/L，4级为ANC<0.5×10^9^/L；血小板减少：3级为PLT<50×10^9^/L且≥25×10^9^/L，4级为PLT<25×10^9^/L。分级判定以移植后每日血常规结果为准。3级或4级细胞减少持续时间计算为自首次达到相应分级阈值之日起至恢复至该分级阈值以上前一日止，单位为天（d）。

6. 粒缺伴发热定义及持续时间：粒缺伴发热定义为在ANC<0.5×10^9^/L期间出现腋温≥38.0 °C（单次）或≥37.7 °C持续≥1 h。粒缺伴发热持续时间按满足上述定义的天数累计计算，至体温恢复且不再满足发热标准之日止。

7. 造血重建标准及随访：连续3 d ANC≥0.5×10^9^/L，首次达标日为中性粒细胞植入时间；不输注血小板情况下，连续7 d PLT≥20×10^9^/L，首次达标日为血小板植入时间。截止随访日期为2025年10月31日，通过查阅电子病历系统信息及电话进行随访。PFS期定义为自体造血干细胞回输日至疾病进展/复发或死亡或随访截止时间，OS期定义为自体造血干细胞回输日至死亡或随访截止时间。

8. 统计学处理：采用SPSS 27.0、Graphpad prism 10.0及R 4.5.2进行统计学分析与绘图。定量资料不符合正态分布者以*M*（*Q*_1_，*Q*_3_）描述，两组间比较采用Mann-Whitney *U*检验，多组连续变量比较采用Kruskal-Wallis *H*检验，分类变量以例数（％）描述，组间比较采用*χ*²检验或Fisher确切概率法。中性粒细胞植入时间及血小板植入时间定义为主要终点，3～4级中性粒细胞减少及血小板减少持续时间、粒缺伴发热持续时间、血小板输注量定义为次要终点，对多重比较采用Benjamini-Hochberg法进行FDR校正并报告*q*值，以*q*<0.05判定差异有统计学意义。采用Logistic回归分析造血植入影响因素，单因素分析中*P*<0.20的变量纳入多因素模型。对临床意义明确且存在潜在共线性的变量［如国际分期系统（ISS）与修订版国际分期系统（R-ISS）］避免同时纳入同一模型。PFS及OS采用Kaplan-Meier法估计并以Log-rank检验比较，多因素生存分析采用Cox回归，双侧*P*<0.05为差异有统计学意义。

## 结果

1. 临床特征：如表1所示，57例接受auto-PBSCT的初诊MM患者，中位年龄57（51，61）岁，男性34例，女性23例。28例采集造血干细胞前化疗次数≤4次、29例>4次。移植前原发病评估达到完全缓解38例，非常好的部分缓解及部分缓解共18例，疾病进展1例。确诊到移植中位时间为339（258，493）d。根据CD34⁺细胞回输剂量分为低剂量组（<4×10^6^/kg，19例）与高剂量组（≥4×10^6^/kg，38例），两组年龄、D-S分期、单克隆免疫球蛋白类型、髓外侵犯等差异均无统计学意义（*P*值均>0.05）。部分患者外院资料缺失或未行细胞遗传学检测，低剂量组患者ISS Ⅲ期（76.5％对35.1％，*P*＝0.005）及R-ISS Ⅲ期（56.2％对20.6％，*P*＝0.012）比例高于高剂量组。此外，高剂量组采集造血干细胞前化疗次数≤4次（60.5％对26.3％，*P*＝0.015）比例更高，移植前化疗次数（6次对8次，*P*＝0.015）以及接受抗CD38单抗治疗比例（10.5％对47.4％，*P*＝0.005）更少。

**表1:** 57例多发性骨髓瘤患者临床特征［例（％）］

临床特征	总体（57例）	低剂量组^a^（19例）	高剂量组^b^（38例）	*χ*^2^/*z*值	*P*值
年龄［岁，*M*（*Q*_1_，*Q*_3_）］	57（51，61）	58（56，64）	57（49，61）	−1.237	0.216
性别				2.332	0.127
男性	34（59.6）	14（73.7）	20（52.6）		
女性	23（40.4）	5（26.3）	18（47.4）		
DS分期（56例）				0.123	0.726
Ⅰ/Ⅱ期	8（14.0）	3（16.7）	5（13.2）		
Ⅲ期	48（86.0）	15（83.3）	33（86.8）		
ISS分期（54例）				7.972	0.005
Ⅰ/Ⅱ期	28（51.9）	4（23.5）	24（64.9）		
Ⅲ期	26（48.1）	13（76.5）	13（35.1）		
R-ISS分期（50例）				6.359	0.012
Ⅰ/Ⅱ期	34（68.0）	7（43.8）	27（79.4）		
Ⅲ期	16（32.0）	9（56.2）	7（20.6）		
M蛋白类型				8.892	0.074
IgG	27（47.4）	9（47.4）	18（47.4）		
IgA	11（19.3）	2（10.5）	9（23.7）		
IgD	3（5.3）	3（15.8）	0（0）		
Kappa轻链型	5（8.8）	2（10.5）	3（7.9）		
Lambda轻链型	10（17.5）	2（10.5）	8（21.1）		
不分泌型	1（1.8）	1（5.3）	0（0）		
髓外侵犯	16（28.1）	7（36.8）	9（23.7）	1.086	0.297
采集造血干细胞前化疗次数≤4次	28（49.1）	5（26.3）	23（60.5）	5.932	0.015
移植前化疗次数［次，*M*（*Q*_1_，*Q*_3_）］	6（5，8）	8（6，10）	6（4.5，7）	−2.431	0.015
采集造血干细胞前使用抗CD38单抗	13（22.8）	9（47.4）	4（10.5）	/	0.005
采集造血干细胞前使用免疫调节剂	48（84.2）	18（94.7）	30（78.9）	1.336	0.248
使用TPO-RA	32（56.1）	14（73.7）	18（47.4）	3.563	0.059
动员方案				2.675	0.469
G-CSF单药	7（12.3）	3（15.8）	4（10.5）		
环磷酰胺+G-CSF	7（12.3）	1（5.3）	6（15.8）		
普乐沙福+G-CSF	18（31.6）	8（42.1）	10（26.3）		
依托泊苷+G-CSF	25（43.9）	7（36.8）	18（47.4）		
确诊到移植时间［d，*M*（*Q*_1_，*Q*_3_）］	339（258，493）	326（237，493）	395（275，613）	−0.990	0.322
移植仓内住院时间［d，*M*（*Q*_1_，*Q*_3_）］	19（18，21）	20（18，21）	19（17，21）	−1.007	0.314
移植前疾病状态				2.470	0.268
CR	38（66.7）	11（57.9）	27（71.1）		
VGPR/PR	18（31.6）	7（36.8）	11（28.9）		
PD	1（1.8）	1（5.3）	0（0）		
MNC［×10^8^/kg，*M*（*Q*_1_，*Q*_3_）］	7.70（4.15，13.29）	7.67（5.00，15.17）	7.72（3.81，13.26）	−0.511	0.610

**注** DS：Durie-Salmon分期；ISS：国际分期系统；R-ISS：修订国际分期系统；M蛋白：单克隆球蛋白；Ig：免疫球蛋白；TPO-RA：血小板生成素受体激动剂；G-CSF：粒细胞集落刺激因子；CR：完全缓解，VGPR：非常好的部分缓解，PR：部分缓解，PD：疾病进展；MNC：单个核细胞；a：CD34⁺细胞回输剂量<4×10^6^/kg；b：CD34⁺细胞回输剂量≥4×10^6^/kg；/：不适用

2. 造血干细胞采集情况：57例MM患者采集的MNC中位数为7.70（4.15，13.29）×10^8^/kg，中位CD34⁺细胞数为5.30（3.49，9.35）×10^6^/kg。其中低剂量组19例，CD34⁺细胞中位数为2.58（2.34，3.48）×10^6^/kg；高剂量组38例，CD34⁺细胞中位数为6.40（5.28，11.82）×10^6^/kg。两组回输MNC、动员方案的差异无统计学意义（*P*值分别为0.610和0.469），见表1。

3. 植入时间：本研究中所有患者均在移植后28 d内获得中性粒细胞及血小板植入，总体中性粒细胞中位植入时间为10.00（9.00，11.50）d，血小板中位植入时间为10.00（9.00，12.50）d。高剂量组中性粒细胞（10.00 d对11.00 d，*P*＝0.014）和血小板（10.00 d对12.00 d，*P*＝0.009）中位植入时间均早于低剂量组，见表2。

**表2:** 多发性骨髓瘤患者不同回输CD34⁺细胞剂量组移植相关并发症、支持治疗与造血重建比较［*M*（*Q*_1_，*Q*_3_）］

观察指标	低剂量组^a^（19例）	高剂量组^b^（38例）	*z*值	*P*值	*q*值
中性粒细胞植入时间（d）	11.00（10.00，11.00）	10.00（9.00，10.00）	−2.445	0.014	–
血小板植入时间（d）	12.00（10.00，13.00）	10.00（8.00，11.25）	−2.600	0.009	–
3级粒细胞减少持续时间（d）	8.00（7.00，9.00）	7.00（6.00，8.00）	−2.464	0.013	0.019
4级粒细胞减少持续时间（d）	7.00（6.00，8.00）	6.00（5.00，7.00）	−2.385	0.016	0.019
3级血小板减少持续时间（d）	14.00（11.00，15.00）	9.00（7.00，11.00）	−3.027	0.002	0.006
4级血小板减少持续时间（d）	8.00（6.00，10.00）	4.00（1.00，7.00）	−3.179	0.001	0.006
粒细胞缺乏伴发热时间（d）	3.00（1.00，5.00）	2.00（0，3.50）	−2.222	0.026	0.026
输注血小板量（U）	4（3，5）	3（2，4）	−2.410	0.015	0.019

**注** ^a^CD34⁺细胞回输剂量<4×10^6^/kg；^b^CD34⁺细胞回输剂量≥4×10^6^/kg。–：中性粒细胞植入时间、血小板植入时间为主要终点，未纳入多重比较校正；其余6项为次要终点，进行多重比较校正，BH-FDR校正：m=6，按Benjamini-Hochberg方法计算并进行单调递增修正，报告*q*值

4. CD34⁺细胞剂量与移植相关并发症的关系：与低剂量组相比，高剂量组≥3级中性粒细胞减少持续时间更短：3级（中位数：7.00 d对8.00 d，*P*＝0.013），4级（中位数：6.00 d对7.00 d，*P*＝0.016）；高剂量组≥3级血小板减少持续时间也更短：3级（中位数：9.00 d对14.00 d，*P*＝0.002），4级（中位数：4.00 d对8.00 d，*P*＝0.001），FDR校正后上述比较*q*值均<0.05，见表2；高剂量组粒缺伴发热持续时间更短（中位数：2.00 d对3.00 d，*P*＝0.026），移植期间血小板输注量更少（中位数：3 U对4 U，*P*＝0.015），FDR校正后*q*值仍<0.05（表2）。

5. CD34⁺细胞剂量与预后：截至末次随访，中位随访296（193，954）d，无移植相关死亡，高剂量组中位PFS与OS均未达到；低剂量组中位PFS期为773（118～1 370）d，中位OS期为812（121～1 432）d。Log-rank检验提示两组PFS与OS差异有统计学意义（*P*值分别为0.040和0.009）。鉴于PFS事件数有限（10例），本研究变量筛选综合以下条件：①研究的核心暴露因素（CD34⁺细胞回输剂量）；②ISS分期及移植前化疗次数在两组基线的差异具有统计学意义（*P*值均<0.05），且为已知的MM预后相关因素的变量，故纳入上述变量以控制主要的潜在混杂偏倚。探索性Cox回归分析结果显示，CD34⁺细胞回输剂量≥4×10^6^/kg与更低的复发风险相关（*HR*＝0.212，95％ *CI*：0.052～0.870，*P*＝0.031）。移植前化疗次数（*HR*＝0.879，95％ *CI*：0.673～1.149，*P*＝0.346）及ISS Ⅲ期（*HR*＝0.880，95％*CI*：0.218～3.550，*P*＝0.858）与PFS未见统计学相关性。

6. 中性粒细胞及血小板植入的影响因素：将患者总体的中位植入时间10 d定义为早期植入界值（≤10 d）。如表3所示，单因素Logistic回归提示，回输CD34⁺细胞剂量≥4×10^6^/kg与中性粒细胞早期植入相关（*OR*＝4.167，95％ *CI*：1.266～13.714，*P*＝0.019），在多因素分析中仍保持独立相关（*OR*＝4.661，95％ *CI*：1.322～16.435，*P*＝0.017）。单因素分析显示回输CD34⁺细胞剂量≥4×10^6^/kg与血小板早期植入相关（*OR*＝4.694，95％ *CI*：1.436～15.350，*P*＝0.011），而ISS Ⅲ期、既往使用抗CD38单抗及移植前化疗次数增加与血小板早期植入受限相关；多因素分析提示ISS Ⅲ期（*OR*＝0.217，95％ *CI*：0.060～0.781，*P*＝0.019）及移植前化疗次数增加（*OR*＝0.706，95％ *CI*：0.520～0.958，*P*＝0.025）与血小板早期植入受限独立相关。

**表3:** 多发性骨髓瘤患者移植后中性粒细胞和血小板早期植入（≤10 d）的单因素分析

因素	中性粒细胞早期植入			血小板早期植入		
	*OR*	95％ *CI*	*P*值	*OR*	95％ *CI*	*P*值
年龄	1.002	0.938～1.070	0.961	1.014	0.953～1.079	0.657
DS分期（Ⅲ期对Ⅰ/Ⅱ期）	2.429	0.532～11.096	0.252	0.714	0.159～3.201	0.660
ISS分期（Ⅲ期对Ⅰ/Ⅱ期）	2.689	0.817～8.854	0.104	0.208	0.065～0.668	0.008
使用抗CD38单抗	0.671	0.184～2.441	0.545	0.254	0.067～0.958	0.043
使用IMiD	0.515	0.120～2.204	0.371	0.328	0.073～1.471	0.145
移植前化疗次数	0.811	0.654～1.005	0.055	0.663	0.500～0.877	0.004
移植前疾病状态（PR+PD对CR+VGPR）	2.500	0.270～23.124	0.419	1.643	0.276～9.788	0.586
CD34⁺细胞剂量（高剂量组对低剂量组）	4.167	1.266～13.714	0.019	4.694	1.436～15.350	0.011

**注** IMiD：免疫调节剂；PR：部分缓解；PD：疾病进展；CR：完全缓解；VGPR：非常好的部分缓解；高剂量组：≥4×10^6^/kg；低剂量组：<4×10^6^/kg

## 讨论

auto-HSCT是适合移植的新诊断MM的标准治疗手段，与传统的美法仑联合类固醇治疗相比，大剂量化疗后联合auto-PBSCT可显著延长患者的无事件生存期以及OS期[2]。关于最佳CD34⁺细胞回输剂量仍是临床决策的争议点。Pasvolsky等[3]的回顾性研究数据显示回输剂量超过2.5×10^6^/kg与更快的造血恢复和更长的PFS期和OS期相关。类似的研究发现，CD34⁺细胞剂量≥5×10^6^/kg时可缩短血小板和粒细胞植入时间，且该剂量与血小板早期植入共同构成生存改善的预测因素[4]。然而，有研究得出不同结论，当回输CD34⁺细胞剂量≥6.50×10^6^/kg，甚至高达≥8×10^6^/kg时，虽可加快造血重建速度，但都未能转化为长期的生存优势[5]–[6]。上述结果提示，CD34⁺细胞剂量与移植结局之间的关系更可能表现为达到一定阈值后有助于促进早期造血恢复，而非简单的线性关系。

本研究高剂量组不仅显著缩短中性粒细胞和血小板植入时间，更能缩短重度骨髓抑制持续时间、减少粒缺伴发热天数及血小板输注需求，这提示优化CD34⁺细胞回输剂量对于降低移植相关并发症、节约医疗资源具有重要临床价值。进一步以“10 d”作为早期植入界值进行分析，结果显示，CD34⁺细胞回输剂量≥4×10^6^/kg是中性粒细胞早期植入的独立相关因素。然而，与中性粒细胞相比，血小板的重建机制显得更为复杂，本研究中ISS Ⅲ期及移植前化疗次数增多与血小板早期植入受限独立相关。有研究发现ISS Ⅲ期患者骨髓中巨核细胞计数显著减少，且可溶性P-选择素、血小板衍生生长因子、丝氨酸等异常增多，抑制巨核细胞的分化与成熟[7]–[8]。另外值得注意的是，移植前化疗次数增加与血小板植入延迟的关联，可能反映了治疗暴露累积及其导致的造血储备下降与动员及采集困难，在auto-HSCT患者中，移植前输血依赖、移植前血小板水平以及回输CD34⁺细胞剂量被证实是延迟植入/延迟恢复的重要预测因子[9]，而诱导治疗周期延长往往更容易伴随治疗相关骨髓抑制、基线血小板偏低或输血需求增加，从而间接导致植入动力学变慢。基于上述发现及现有实践建议，移植候选MM患者的诱导治疗应在获得充分缓解的同时，尽量避免不必要的诱导治疗周期延长，对于预计动员风险较高或诱导治疗周期较多者，可提前制定更积极的动员策略，并在移植时确保足够的CD34⁺细胞回输，以减少因血小板植入延迟的输血支持负担。

近年来含抗CD38单抗在内的四药诱导方案可提高深度缓解率，但多药联合方案的使用对患者CD34⁺细胞采集量可能会产生不同程度的影响。在临床试验及真实世界研究中均发现，诱导方案中包含抗CD38单抗时，动员后采集的CD34⁺细胞剂量相对更低[10]–[11]，造血干细胞动员效能减低可能与黏附相关基因如JCAD、NRP2、MDK等潜在靶基因过表达有关，而非效应机制对CD34⁺细胞的杀伤[12]。本研究中也观察到低剂量组较高剂量组采集造血干细胞前治疗次数更多、既往抗CD38单抗暴露比例更高，而抗CD38单抗暴露与更慢的血小板恢复有一定相关性，上述结果提示CD34⁺回输剂量可能部分反映患者治疗暴露与动员难度差异，考虑高危患者对串联移植的需求，抗CD38单抗对采集CD34⁺细胞数量的影响需要引起关注，此类患者可能需要针对性地使用阻断黏附的药物如CXCR4拮抗剂普乐沙福来克服抗CD38单抗治疗带来的动员困难。另一方面，BCMA CAR-T等细胞免疫疗法带来的免疫效应细胞相关血液毒性（ICAHT）逐渐成为管理热点，有研究证实自体造血干细胞回输可有效缓解此类血细胞减少[13]，因此，在初诊患者首次动员中争取更高采集量具有潜在价值，但本队列未保留额外冻存，未来研究可评估储备策略。

关于生存结局，本研究在有限随访期内观察到高剂量组与更优的PFS和OS相关，对于PFS，在纳入ISS分期及移植前化疗次数后，较高CD34⁺细胞回输剂量仍与较好的PFS相关，但由于OS事件极少、PFS事件数有限，相关结果仍应作为探索性发现谨慎解读。既往有研究提出auto-HSCT后第15天的绝对淋巴细胞计数（ALC-15）是MM和非霍奇金淋巴瘤患者生存的独立强预后因素[14]，同时，也有研究认为移植物中的绝对淋巴细胞剂量（A-ALC）与ALC-15恢复及生存结局有关[15]，然而，在本研究中，由于回顾性资料限制，仅有19例具有可用的ALC-15数据，且事件数过少，无法进一步评估其与预后的关系；此外，仅44例具备移植物A-ALC数据，补充分析显示回输CD34⁺细胞剂量与回输淋巴细胞剂量之间未见相关性。因此，本研究虽观察到较高CD34⁺细胞回输剂量与较好生存结局相关，但尚不能从早期淋巴细胞恢复或移植物淋巴细胞成分角度对其作出解释。此外需要指出的是，CD34⁺细胞计数主要反映移植物数量，不能完全替代质量评价。受回顾性资料限制，本研究缺乏CD34^+^细胞活性及功能学指标，故结果应作为相关性证据解读。

综上所述，初诊MM患者CD34⁺细胞回输剂量≥4×10^6^/kg与移植后更快的造血重建及更少的移植并发症相关，其中CD34⁺细胞回输剂量≥4×10^6^/kg是10 d内中性粒细胞植入的独立相关因素，ISS Ⅲ期及移植前化疗次数增多是10 d内血小板植入手段独立相关因素。CD34⁺细胞回输剂量≥4×10^6^/kg可能带来更优的PFS和OS。鉴于本研究为单中心回顾性分析，且样本量有限、基线存在一定差异，尤其生存事件数较少，相关生存结论仍需在更大样本、随访更充分并进一步校正混杂因素的研究中加以验证。
